# Endobronchial Metastasis of Renal Carcinoma: A Case Report and Review of Previous Literature

**DOI:** 10.3389/fsurg.2021.658749

**Published:** 2021-05-21

**Authors:** Guang-Lei Zhang, Shu Chen, Jin-Dong Li, Chun-Guang Wang

**Affiliations:** Department of Thorax, Second Hospital of Jilin University, Changchun, China

**Keywords:** endobronchial metastasis, diagnosis, treatment, renal cell carcinoma, surgical therapy

## Abstract

The definition of endobronchial metastasis (EBM) lacks clarity because it is currently based on the judgments of surgeons; it is rare in patients with nonpulmonary malignancies. Although EBM represents an advanced stage of malignancy, it does not necessarily indicate a poorer prognosis than that for its primary tumors. The present study defines EBM as bronchoscopy-visible lesions with histologically confirmed primary extrapulmonary tumors, excluding those primary lung tumors with involvement of the bronchial lumen. A bronchoscopy and biopsy provide strong proof for diagnosis. Complete surgical resection is the best choice for patients with EBM. This study analyzed the case of a 69-year-old male patient who had undergone a radical left nephrectomy several years previously after the identification of a bronchoscopy-visible lesion in the left main bronchus. The lesion was initially diagnosed as an angiogenic tumor but was eventually confirmed by surgical biopsy as EBM from the left kidney. After diagnosis, the patient underwent a left pneumonectomy. The analysis of this case focused on diagnosis, symptoms, radiographic findings, treatment, and prognosis. A review of the previous literature relating to EBM was also conducted.

## Introduction

Although lung metastasis from renal carcinoma represents 45% to 76% of all metastatic renal cell carcinoma cases (1, 2), endobronchial metastasis (EBM) from the kidney is rare. A study of autopsies performed on 109 patients with extrapulmonary malignancies (3) found that only 20% of cases of metastatic lung carcinoma from extrapulmonary carcinoma present definitive infiltration of the bronchus. Another study, in which the autopsies of 1,359 patients were analyzed, found that involvement of the main airway was present only in 2 to 5% of patients with metastatic solid malignancies (4).

Although EBM is an advanced staging manifestation of extrapulmonary carcinoma, it does not necessarily indicate a poor prognosis as demonstrated by previous serious case series and autopsies (5). The prognosis of patients with EBM depends on the subtype of the primary tumor, the presence of other metastatic sites, the involvement of the hilar or mediastinal lymph nodes, and complete surgical resection (1, 6–12). The interval between the diagnosis of primary tumors and of EBM varies widely but is mostly between 50 and 60 months. Sorensen (3) and Akoglu (13) report the mean survival period after diagnosis of EBM as 15 and 16 months, respectively, and Coriat (7) and Akoglu (13) report the median survival period as 18.9 and 27 months, respectively, which may indicate a poor survival time (one to 2 years for most patients).

Pathological examination is the gold standard for the diagnosis of EBM, and despite the high false-negative rate for diagnosing EBM seen in the following case report, a bronchoscopy with biopsy is more useful than other procedures. Other procedures that can be used for the diagnosis of EBM include radiography, computed tomography (CT), endobronchial ultrasound-guided transbronchial needle aspiration (EBUS-TBNA), positron emission tomography–computed tomography (PET–CT), and bronchial brushing (14). The management and treatment of patients with EBM from extrapulmonary malignancies are determined by the characteristics of the primary tumors, the anatomical location of the lesions, evidence of other metastatic sites, and performance status (15–17).

The present study analyzed the case of a 69-year-old male patient with endobronchial metastasis from the left kidney who had undergone radical resection several years previously. The objective was to identify the patient's related conditions. A review of the previous literature pertaining to EBM was also conducted.

## Case Report

A 69-year-old male patient who had been experiencing a cough, expectoration, and hemoptysis for a month was admitted to the thoracic surgery department. The patient had undergone resection of the left renal mass many years previously, and postoperative pathology showed clear cell renal cell carcinoma. The patient had not received any neoadjuvant therapy or adjuvant therapy before or after the operation. Until the onset of the above symptoms, the patient had not received systematic follow-up or any imaging observation.

**Figure 1:**
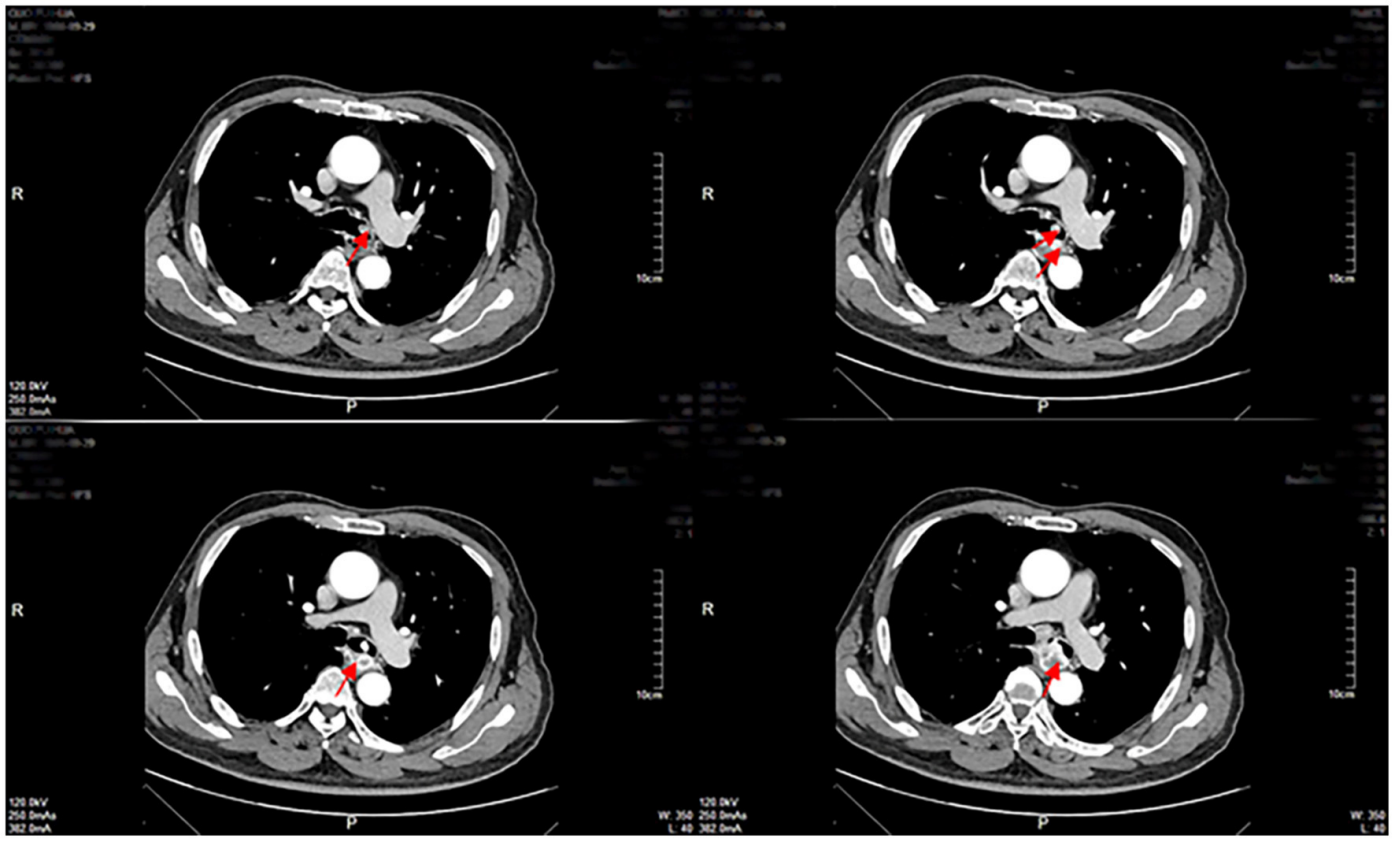
Enhanced CT examinations revealed a mass of inhomogeneous shadow located in the left main bronchus close to the carina as shown by the arrows.

**Figure 2:**
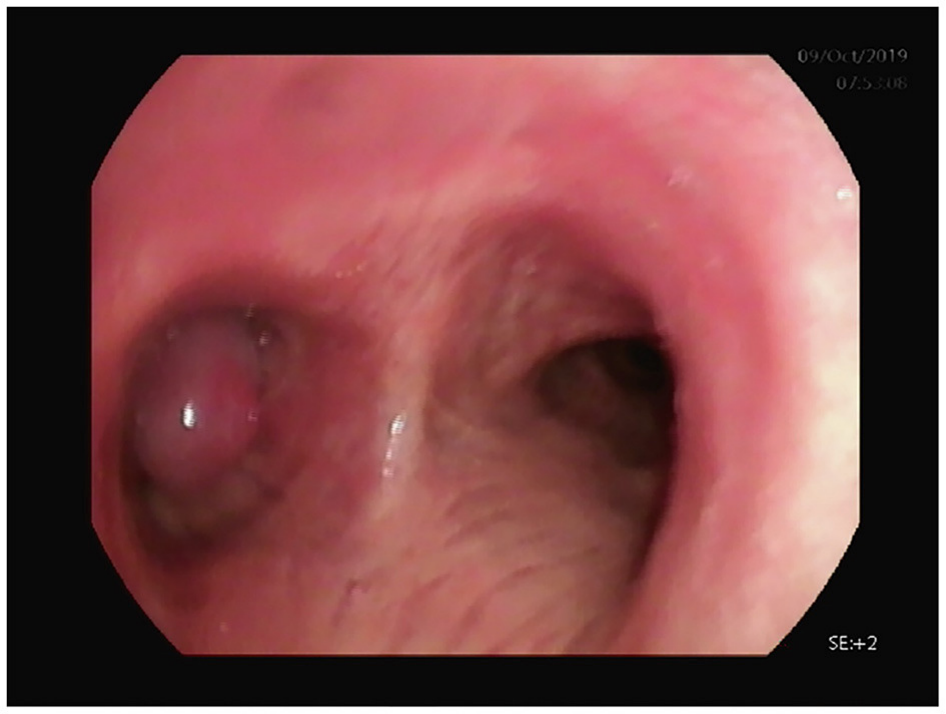
Bronchoscopy examination revealed a left main bronchus embolism by a sarcomatoid mass.

Enhanced CT revealed a large irregular inhomogeneous lesion presenting as an obstructive mass at the origin of the left main bronchus (Figure 1). A bronchoscopy with biopsy indicated a new angiogenic tumor occluding the left main bronchus (Figure 2). The lesion was initially diagnosed as a common hemangioma with a high risk of hemorrhage and bronchial embolism. Therefore, urgent surgery was performed.

After the operation, the pathological findings for the tumor were as follows: Clear cell nest infiltration was found under the bronchial mucosa, and the cells were heteromorphic and infiltrated into the surrounding smooth muscle tissue; clear cell nests were found in the bronchial wall of the left lung, cancer infiltration was found in the vessels, no tumor was found at the cutting edge of the bronchus or blood vessels, squamous metaplasia was found in the local bronchial mucosa, chronic inflammatory cell infiltration was found in other lung tissues, and no cancer metastasis was found in the lymph nodes (0/4). The immunohistochemical staining results were as follows: The tumor was positive (+) for CD10, CAIX, CK (AE1/AE3) (partial +), Ki67 (+ rate 20%), Pax-8 (partial, weak +), and vimentin; it was negative for TTF-1, CK7, CD117, TFE3, S-100, HMB45, and napsin A. The TNM stage was T2N0Mx.

Thus, the biopsy indicated a clear-cell renal carcinoma that was identical to the previously identified left renal carcinoma. A diagnosis of EBM from the left kidney was finally established through surgical biopsy. It is accepted that renal carcinoma metastasis often indicates a hypervascular tumor with a high risk of massive hemorrhage, which, although being the most dangerous complication of EBM, is also its least common. The patient underwent a radical left pneumonectomy to resection the lesion and distal lung tissue. No metastasis was found on PET–CT after the left pneumonectomy. The patient's 6-min walk distance was 350 meters.

Postoperative therapy and management were individualized and included antibiotic and symptomatic therapy for several days. After being discharged from the hospital, the patient showed no further symptoms and had no significant radiographic manifestations other than the left movement of the mediastinum. Palliative therapies, such as chemotherapy and radiotherapy, are essential for most patients with EBM, so this patient also underwent chemotherapy. Sunitinib, an agent targeting vascular endothelial growth factor receptor (VEGFR), can also be used to treat advanced renal cell carcinoma. According to previous studies, however, this agent is associated with an antiangiogenic effect, which may lead to bronchial fistulas and other nonspecific symptoms, such as asthenia, diarrhea, abdominal pain, hypothyroidism, hemorrhage, and fistulas of other organs (18, 19). Due to the adverse effects and limited therapeutic effect of sunitinib, it was not given to the patient in this study. Radiological follow-up of the patient included radiography and CT examinations, which were performed every 6 months for several years. This study was conducted in accordance with the Declaration of Helsinki. It was approved by the Ethics Committee of the Second Hospital of Jilin University, and informed consent was taken from all the patients.

## Discussion

The diagnosis modalities of EBM vary and include bronchoscopy with biopsy, surgical biopsy, CT, X-rays, EBUS-TBNA, and bronchoalveolar lavage. Bronchoscopy with biopsy is the most effective diagnostic method for patients with EBM, but because the false-negative rate with this method is high, its diagnostic yield in evaluating EBM is low. In the case of the patient in the current case study, however, a bronchoscopy provided strong evidence of EBM. Although surgical biopsy provides a higher diagnostic yield than bronchoscopy with biopsy, its higher price, lower operability, and greater trauma limit its application in daily clinical work. Other diagnosis modalities, such as radiographic examination, bronchial brushing, and EBUS-TBNA, can be seen as auxiliary diagnosis modalities (although some studies and case reports emphasize that the utility of these modalities is underestimated).

The distinction between EBM and bronchogenic tumors is not clear and requires immunohistochemistry and genetic knowledge. In the patient in the current case report, genetic and molecular biological examination identified a loss of genetic material involving chromosome 3p and somatic mutation in the von Hippel–Lindau tumor suppressor gene (1). However, the limitations of relevant techniques hinder their clinical use. Instead, the pathological identification of endobronchial lesions and primary tumors via bronchoscopy with biopsy or surgical biopsy provides sufficient proof for an EBM diagnosis for most clinicians. It is, therefore, important that clinicians are fully aware of EBM when diagnosing patients with a medical history of extrathoracic tumors even if those patients have undergone complete surgical resection of the primary tumors.

The most common symptoms of EBM are coughing and hemoptysis, followed by dyspnea and wheezing (6, 13). This is consistent with the symptoms of the patient in the current case report. Although the above symptoms are not exclusive to patients with EBM, the appearance of any one of them in a patient with extrapulmonary tumors may indicate the possibility of EBM. According to Kiryu et al. (17), Heitmiller (20), and Poe (21), however, asymptomatic patients account for 62.5, 52, and 62%, respectively, of all cases. Therefore, the use of bronchoscopy with biopsy is sensible for patients with histologically identified malignant extrapulmonary tumors presenting with corresponding symptoms.

Radiographic findings include single and multiple nodules, atelectasis, effusion, and hilar or mediastinal lymphadenopathy (13). In 13 cases presented by Akoglu (13), all 21 of the main lesions were located in the right bronchi as were 20 main lesions presented by Kiryu et al. (17). The reason for the high frequency of lesions being located in the right bronchi is not clear. The relationship between the numbers of primary site cases and survival months is summarized in Table 1.

**Table 1:** The relationship between the number of primary site cases and survival months.

	**Renal**		**Breast**		**Colorectal**	
	***n***	**Survival**	***n***	**Survival**	***n***	**Survival**
Sorensen	34	20	72	15	30	15
Akoglu	2	12	3	13	2	35
Coriat	–	–	–	–	7	55
Kiryu	–	–	3	11.0	6	8.7

The management and treatment of patients with EBM should be individualized based on primary tumors, location and number of secondary tumors, and performance status. For patients with advanced-stage EBM with multiple metastatic lesions or locations, palliative treatment, such as diathermic snares, grasping with forceps under rigid bronchoscope (22), endobronchial radiation (brachytherapy), photodynamic therapy, electrocoagulation, prosthetic stents, intratumoral ethanol injections, and Nd: Yag laser debulking therapy are available. These treatments aim to promote quality of life rather than cure the patient. Radical surgery is the most effective method for curing or extending the survival period of patients with early-stage EBM with a single lesion or location. Surgical therapies, such as pneumonectomy and lobectomy, are also suitable for EBM patients with confined metastatic lesions.

There are several favorable prognostic factors and surgical signs for patients with EBM, including fewer than seven metastases, lack of hilar or mediastinal nodal involvement, pulmonary metastases smaller than 4 cm, and confinement to a single lung (1). Total surgical resection is beneficial even for patients who do not have complete surgical signs (1); although there is no strict indication for operation, these patients will benefit to some extent. Postoperative bronchial fistula can easily occur after pneumonectomy. The tension is reduced if the broken end of the trachea is close to the carina of bronchus. In the current case, the distance between the broken end of the trachea and the carina led to high tension, which is a high-risk factor for postoperative bronchial fistula. Because of the serious adverse effects associated with bronchial fistula of therapeutic drugs, such as sunitinib and pazopanib, the use of sunitinib is uncommon for patients with EBM in our hospital (18, 19).

Some emergency situations, such as acute tumor embolism or massive bleeding, require specially targeted therapy. The recanalization of a bronchial cavity occluded by a tumor mass or blood clots involves grasping with forceps under a rigid bronchoscope (22) or bronchial artery embolization (BAE) (16) before an interventional bronchoscopy (including laser resection and stent placement); however, the most useful procedure for dealing with a tumor embolism is urgent surgery. Massive bleeding is common in hypervascular tumors, such as renal cell carcinomas, and in these cases, BAE can be performed as a palliative method instead. In the patient in the present case report, various complications were identified after surgery, including atelectasis, pneumothorax, massive bleeding, and wheezing; such complications have also been recorded in previous case reports. Symptomatic treatment is the most effective treatment method for these common complications.

The main endobronchial therapies include laser therapy, cryotherapy, electrosurgical snare, argon plasma coagulation (APC), stent, bronchoscopy, and mechanical resection. Endobronchial therapy is not a good treatment for EBM patients as such therapy generally aims to relieve symptoms (mainly those associated with a tumor blocking the airway) without aiming to cure. It is suggested that endobronchial therapy for EBM-induced airway stenosis should be carried out after metastatic resection; however, there are risks of bronchial fistula, insufficiently open airway, asphyxia, and mediastinal emphysema. Serious complications of endobronchial therapy have been reported. In the vast majority of EBM patients, surgical treatment is the first choice (23, 24).

Breast, colon, and renal carcinomas are reported as being most often associated with EBM (25). Although EBM represents an advanced stage of malignancy, it does not necessarily indicate a poorer prognosis than that for malignant tumors without EBM. Analysis of the present case report and previous literature shows that the definition of the survival period for patients with EBM and those with comparable confined malignant tumors is not clear (5, 7, 23). Prognosis depends on the subtype of the primary tumor(s), the presence of other metastatic sites, the involvement of the hilar or mediastinal lymph nodes, and complete surgical resection. However, clear cell renal cell carcinoma represents 70 to 75% of cases at surgical resection (1) and indicates a poorer prognosis than other subtypes of renal cell carcinoma, such as papillary and chromophobe renal cell carcinoma. Furthermore, complete surgical resection seems to an independent favorable prognostic factor.

## Conclusion

The incidence of EBM from extrathoracic tumors is lower than that of pulmonary metastasis. Although EBM represents an advanced stage of malignancy, it does not necessarily indicate a poorer prognosis than that for primary tumors without EBM. It is difficult for clinicians to differentiate between EBM and bronchogenic tumors when immunohistochemistry and genetic examination are not available. A diagnosis of EBM is established when the pathological identity of bronchial lesions and primary tumors is determined by bronchoscopy with biopsy in the context of a medical history of primary tumors. Treatment should be individualized, and complete surgical resection is the most effective treatment in most cases.

## Data Availability Statement

The original contributions presented in the study are included in the article/supplementary material, further inquiries can be directed to the corresponding authors.

## Author Contributions

G-LZ: conception and design of the research and analysis and interpretation of the data. SC: acquisition of data and statistical analysis. J-DL: obtaining financing and writing of the manuscript. C-GW: critical revision of the manuscript for intellectual content. All authors contributed to the article and approved the submitted version.

## Conflict of Interest

The authors declare that the research was conducted in the absence of any commercial or financial relationships that could be construed as a potential conflict of interest.
